# Supplementary material for: Tumor Growth Suppression of Pancreatic Cancer Orthotopic Xenograft Model by CEA-Targeting CAR-T Cells
Source: Cancers (Basel). 2023 Jan 18;15(3):601. doi: 10.3390/cancers15030601 (PMC9913141; doi:10.3390/cancers15030601)
Supplement: Supplementary file 1 [file cancers-15-00601-s001.zip › cancers-2078668-supplementary.pdf]

Supplementary Figure S1.

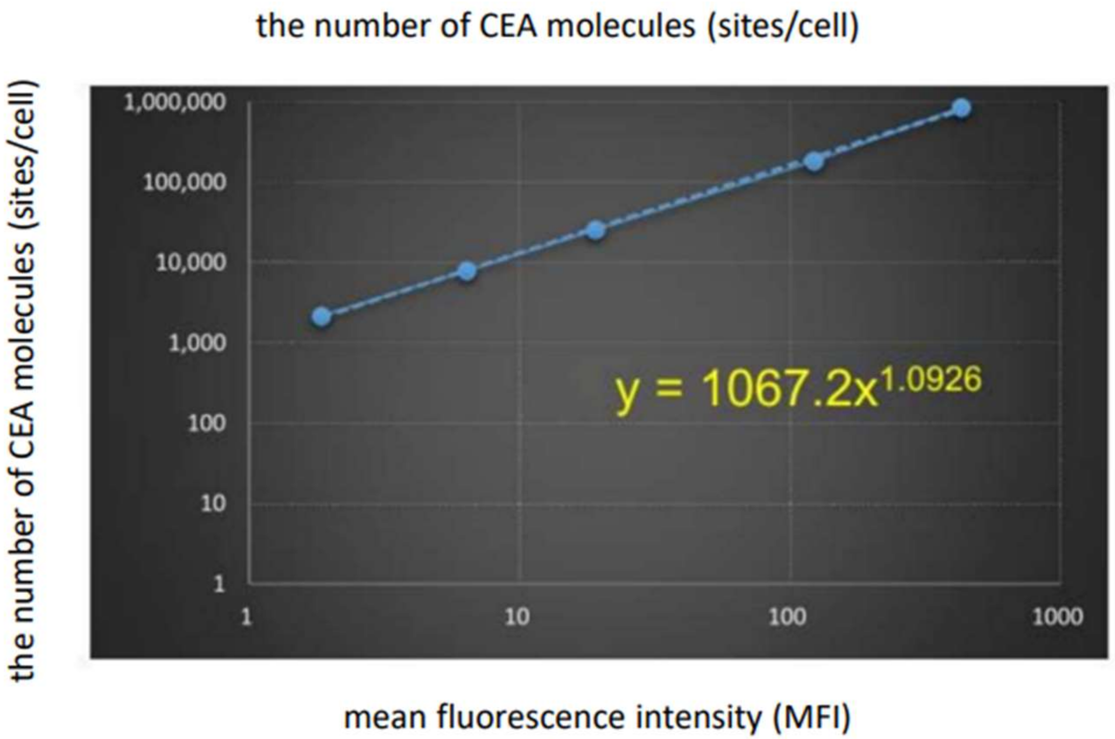

Calibration curve showing MFI versus the number of CEA molecules on the cell (log-log scale) for the 5 Calibration Bead populations by QIFIKIT.

Supplementary Figure S2.

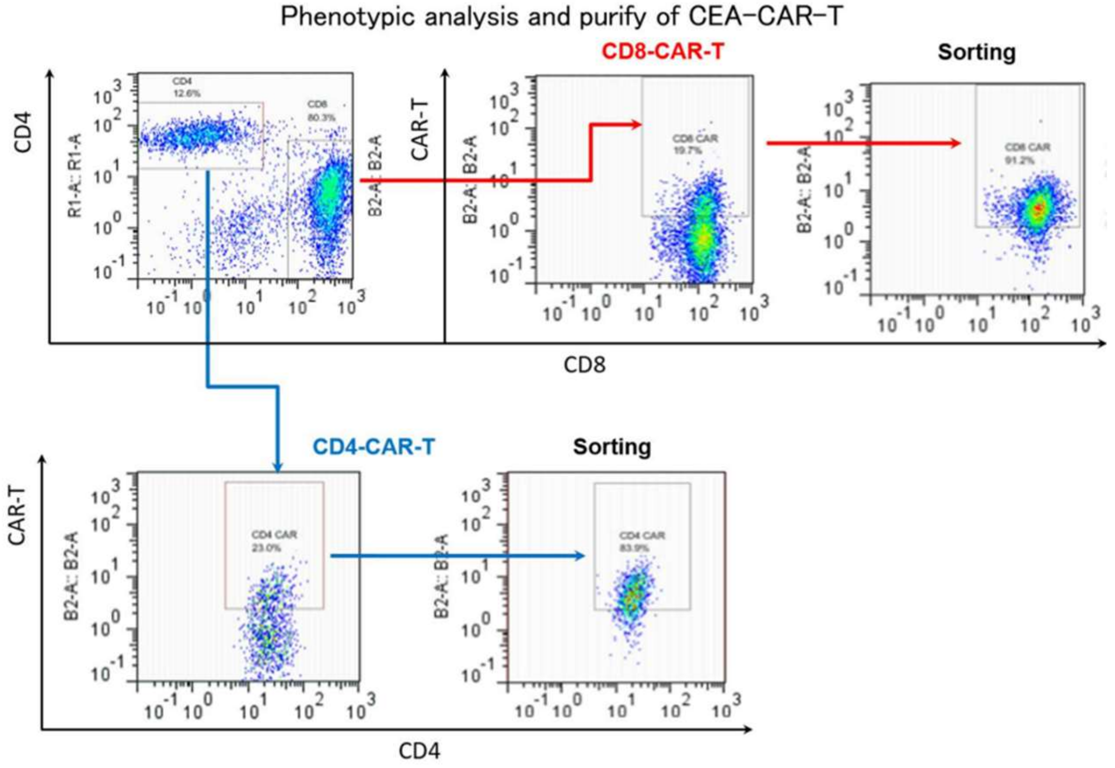

Both CD4^{+} and CD8^{+} T cells were modified with CAR and used for adoptive transfer in this study. CAR expression was monitored by flow cytometry using APC-conjugated antibody-CD4, PE-Cy5 conjugated anti-CD8, and PE-conjugated streptavidin anti-biotinylated recombinant CEA. CAR-Ts were isolated by magnetic cell separators using anti-biotin microbeads.
